# The genome sequence of the spectacle, *Abrostola tripartita *Hufnagel, 1766

**DOI:** 10.12688/wellcomeopenres.17355.1

**Published:** 2021-12-06

**Authors:** Douglas Boyes, Liam Crowley, Peter W.H. Holland, University of Oxford and Wytham Woods Genome Acquisition Lab, Darwin Tree of Life Barcoding collective, Wellcome Sanger Institute Tree of Life programme, Wellcome Sanger Institute Scientific Operations: DNA Pipelines collective, Tree of Life Core Informatics collective, Darwin Tree of Life Consortium

**Affiliations:** 1 UK Centre for Ecology & Hydrology, Wallingford, UK; 2 Department of Zoology, University of Oxford, Oxford, UK

**Keywords:** Abrostola tripartita, the spectacle, genome sequence, chromosomal, Lepidoptera

## Abstract

We present a genome assembly from an individual male *Abrostola tripartita *(the spectacle; Arthropoda; Insecta; Lepidoptera; Noctuidae). The genome sequence is 381 megabases in span. The majority of the assembly (99.99%) is scaffolded into 31 chromosomal pseudomolecules, with the Z sex chromosome assembled.

## Species taxonomy

Eukaryota; Metazoa; Ecdysozoa; Arthropoda; Hexapoda; Insecta; Pterygota; Neoptera; Endopterygota; Lepidoptera; Glossata; Ditrysia; Noctuoidea; Noctuidae; Plusiinae; Abrostola; *Abrostola tripartita* Hufnagel, 1766 (NCBI:txid938171).

## Background

*Abrostola tripartita* (the spectacle) is a grey and white noctuid moth recorded from across the Palaearctic region. It is found commonly across the UK where it has increased significantly in abundance in recent decades ( Sterling & Henwood, 2020). The common name derives from two rings of grey hairs on the thorax with the appearance of a pair of goggles, visible when the moth is viewed from anterior; these hairs are on the thorax and are not associated with the head or eyes. The larvae feed on nettle ( *Urtica dioica*) and may be found across any habitat where the food plant is in abundance. Adults often feed at flowers including red valerian ( *Centranthus ruber)* and sage ( *Salvia*). In the UK the adult flight period occurs as a single generation in the north (May to July) and as two generations in the south (May-July and July-September) ( Waring *et al.*, 2003). Overwintering occurs as a pupa among plant litter on the ground or under bark.

## Genome sequence report

**Figure 1.**
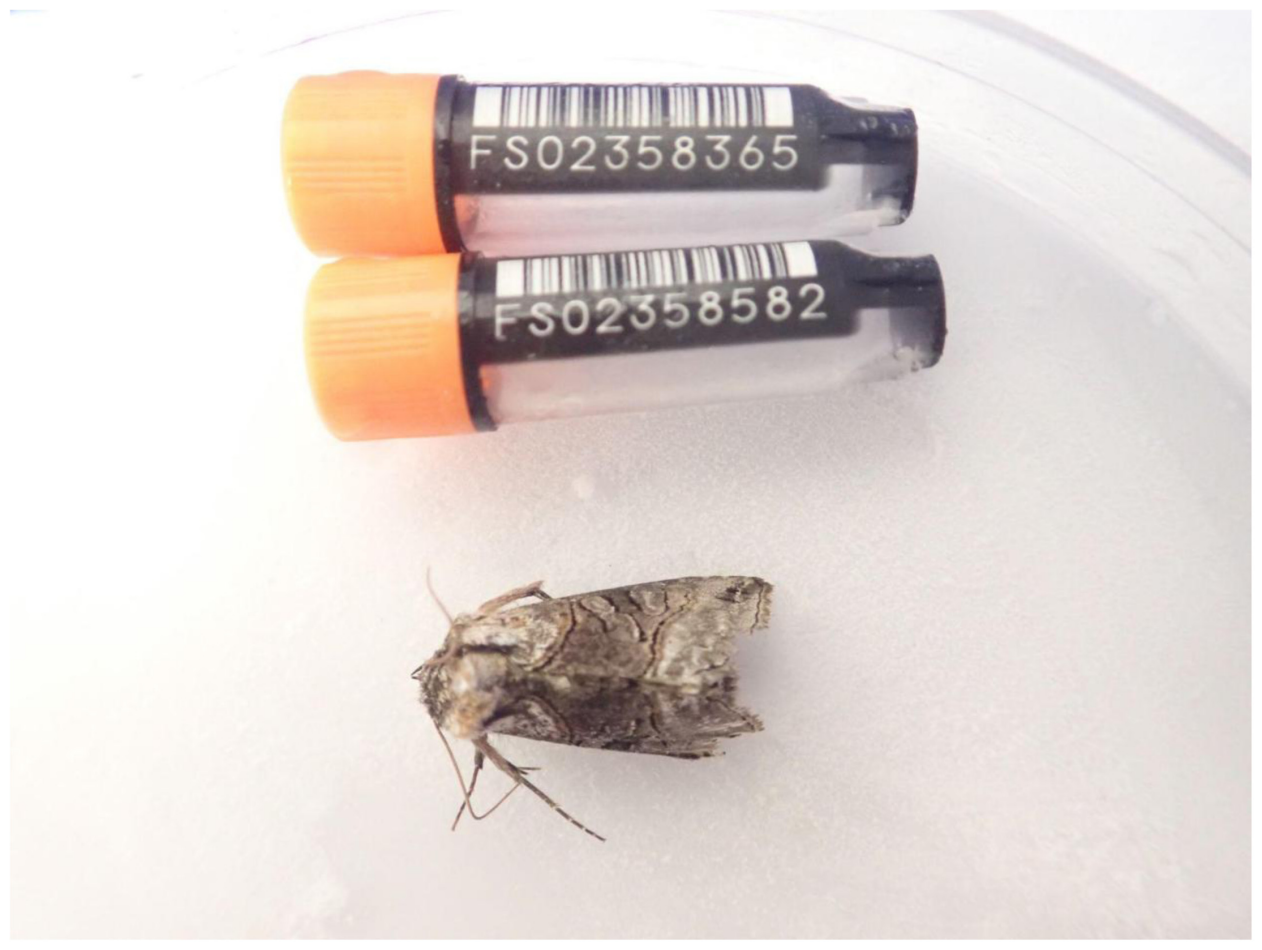
Image of the ilAbrTrip1 specimen taken prior to preservation and processing. Specimen shown next to FluidX storage tube, 43.9 mm in length.

The genome was sequenced from one male *A. tripartita* ( Figure 1) collected from Wytham Woods, Oxfordshire (biological vice-county: Berkshire), UK (latitude 51.769, longitude -1.339). A total of 54-fold coverage in Pacific Biosciences single-molecule long reads and 102-fold coverage in 10X Genomics read clouds were generated. Primary assembly contigs were scaffolded with chromosome conformation Hi-C data. Manual assembly curation corrected 30 missing/misjoins and removed 3 haplotypic duplications, reducing the assembly size by 0.44% and the scaffold number by 20.00%.

**Figure 2.**
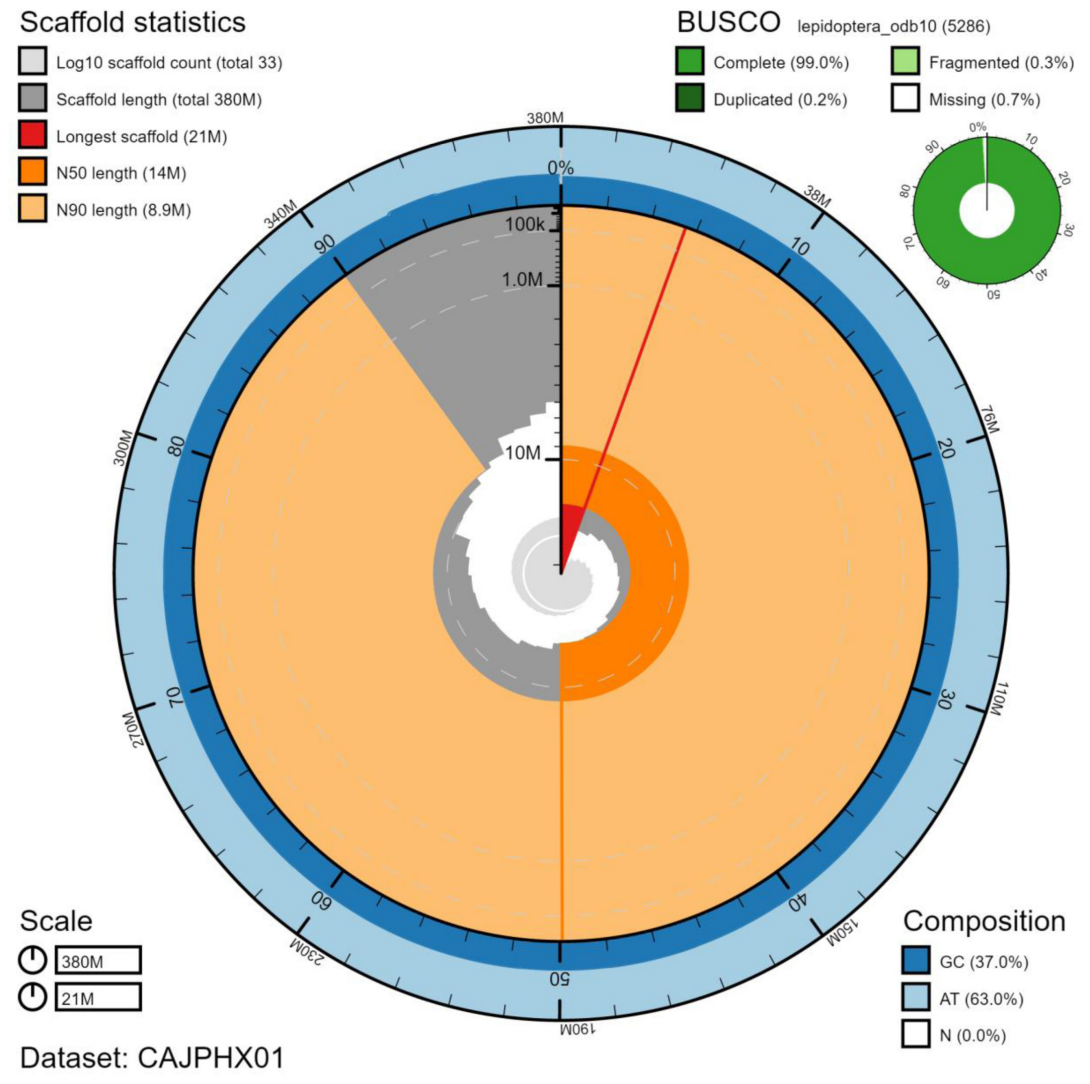
Genome assembly of *Abrostola tripartita*, ilAbrTrip1.1: metrics. The BlobToolKit Snailplot shows N50 metrics and BUSCO gene completeness. The main plot is divided into 1,000 size-ordered bins around the circumference with each bin representing 0.1% of the 381,057,457 bp assembly. The distribution of chromosome lengths is shown in dark grey with the plot radius scaled to the longest chromosome present in the assembly (20,963,991 bp, shown in red). Orange and pale-orange arcs show the N50 and N90 chromosome lengths (13,645,312 and 8,916,110 bp), respectively. The pale grey spiral shows the cumulative chromosome count on a log scale with white scale lines showing successive orders of magnitude. The blue and pale-blue area around the outside of the plot shows the distribution of GC, AT and N percentages in the same bins as the inner plot. A summary of complete, fragmented, duplicated and missing BUSCO genes in the lepidoptera_odb10 set is shown in the top right. An interactive version of this figure is available at https://blobtoolkit.genomehubs.org/view/ilAbrTrip1.1/dataset/CAJPHX01/snail.

The final assembly has a total length of 381 Mb in 32 sequence scaffolds with a scaffold N50 of 13.6 Mb ( Table 1). The majority of the assembly sequence (99.99%) was assigned to 31 chromosomal-level scaffolds, representing 30 autosomes (numbered by sequence length), and the Z sex chromosome ( Figure 2– Figure 5; Table 2). The assembly has a BUSCO v5.1.2 ( Manni *et al.*, 2021) completeness of 99.0% (single 98.8%, duplicated 0.2%) using the lepidoptera_odb10 reference set. While not fully phased, the assembly deposited is of one haplotype. Contigs corresponding to the second haplotype have also been deposited.

**Table 1.** Genome data for *Abrostola tripartita*, ilAbrTrip1.1.

*Project accession data*	
Assembly identifier	ilAbrTrip1.1
Species	*Abrostola tripartita*
Specimen	ilAbrTrip1
NCBI taxonomy ID	NCBI:txid938171
BioProject	PRJEB43740
BioSample ID	SAMEA7520667
Isolate information	Male, thorax (genome assembly), head (Hi-C), abdomen (RNA-Seq)
*Raw data accessions*	
PacificBiosciences SEQUEL II	ERR6412364
10X Genomics Illumina	ERR6054553-ERR6054556
Hi-C Illumina	ERR6054552
Illumina polyA RNA-Seq	ERR6054557
*Genome assembly*	
Assembly accession	GCA_905340225.1
Accession of alternate haplotype	GCA_905340255.1
Span (Mb)	381
Number of contigs	34
Contig N50 length (Mb)	13.6
Number of scaffolds	32
Scaffold N50 length (Mb)	13.6
Longest scaffold (Mb)	16.0
BUSCO * genome score	C:99.0%[S:98.8%,D:0.2%],F:0.3%,M:0.7%,n:5286

*BUSCO scores based on the lepidoptera_odb10 BUSCO set using v5.1.2. C= complete [S= single copy, D=duplicated], F=fragmented, M=missing, n=number of orthologues in comparison. A full set of BUSCO scores is available at https://blobtoolkit.genomehubs.org/view/ilAbrTrip1.1/dataset/CAJPHX01/busco.

**Figure 3.**
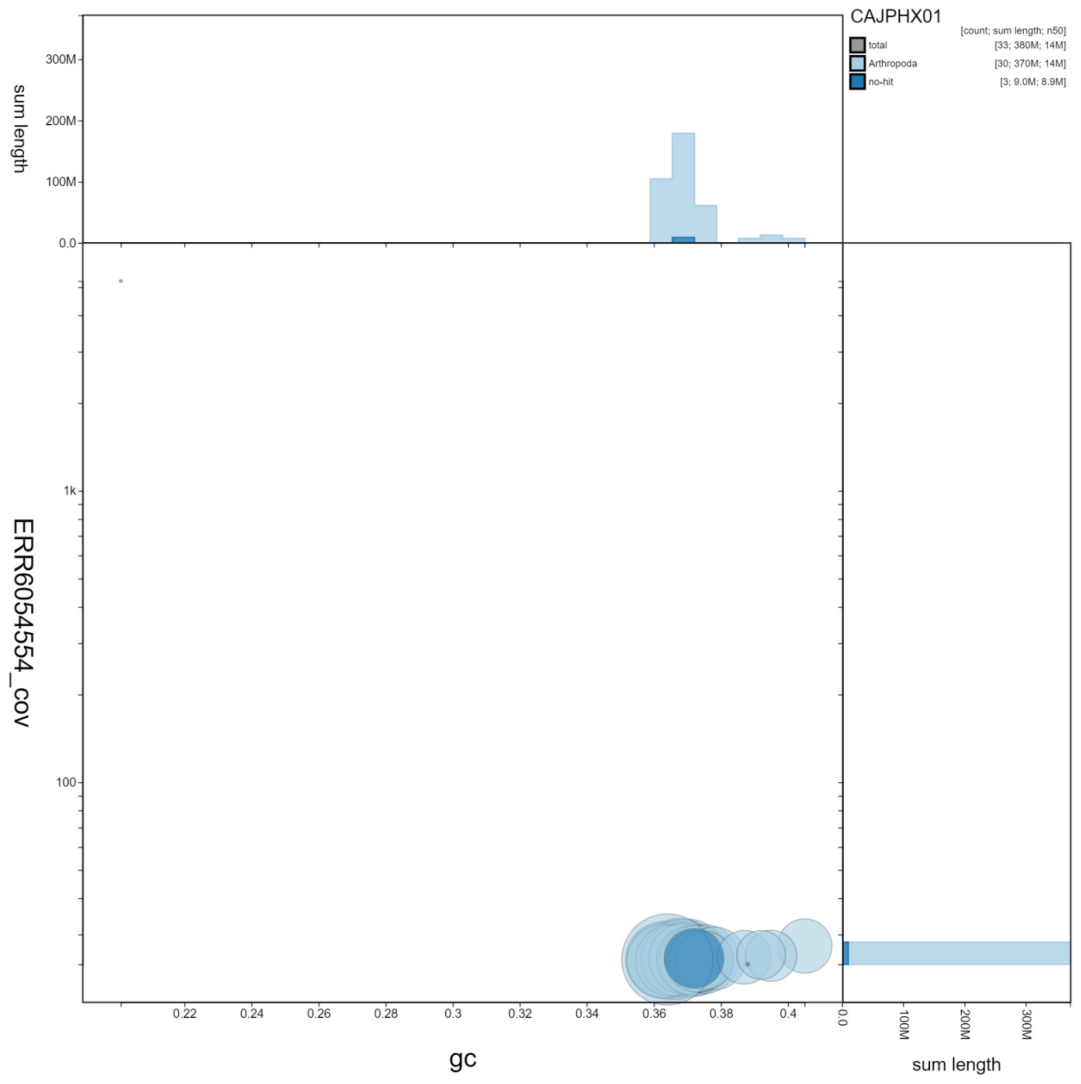
Genome assembly of *Abrostola tripartita*, ilAbrTrip1.1: GC coverage. BlobToolKit GC-coverage plot. Scaffolds are coloured by phylum. Circles are sized in proportion to scaffold length Histograms show the distribution of scaffold length sum along each axis. An interactive version of this figure is available at https://blobtoolkit.genomehubs.org/view/ilAbrTrip1.1/dataset/CAJPHX01/blob.

**Figure 4.**
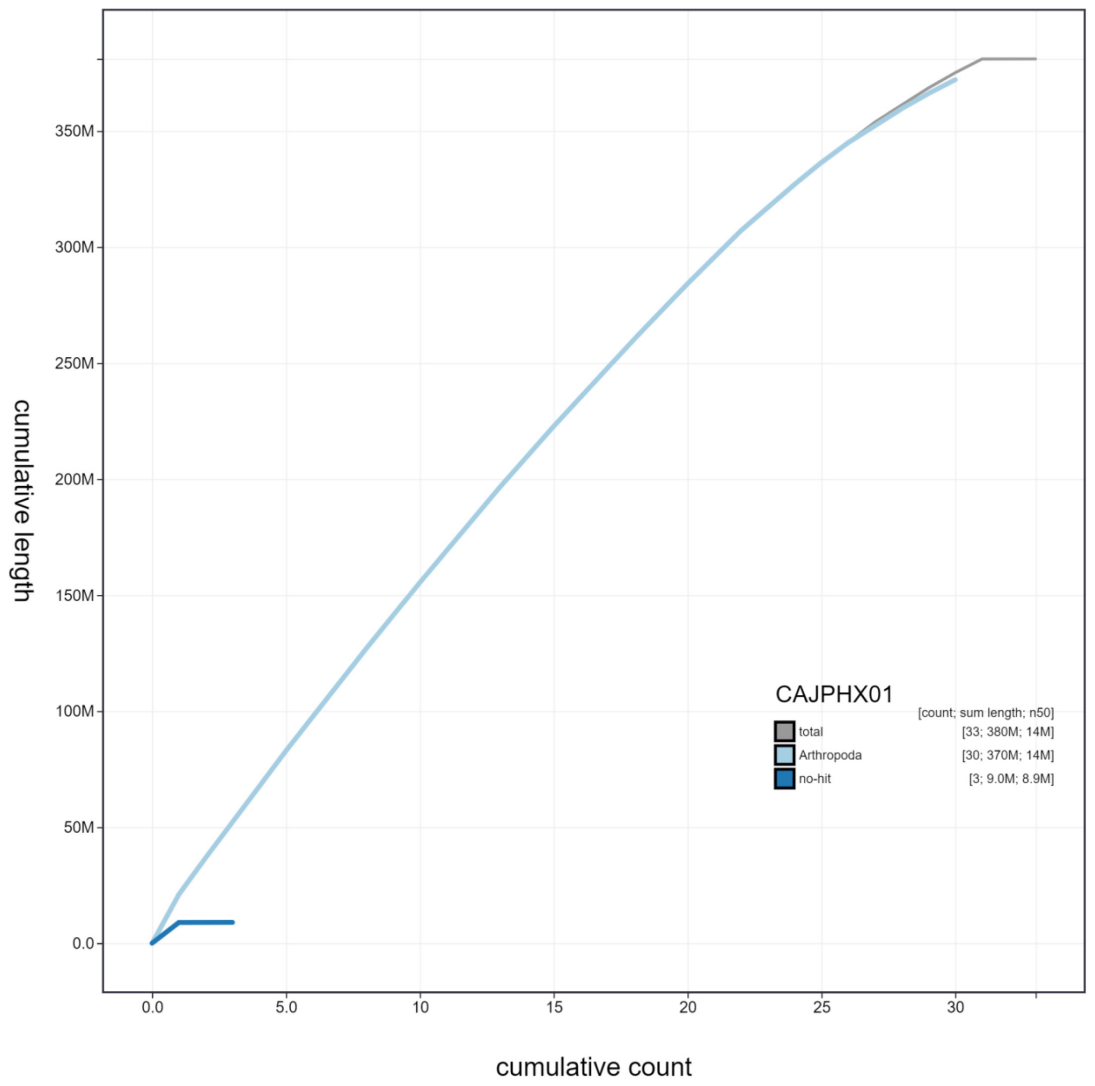
Genome assembly of *Abrostola tripartita*, ilAbrTrip1.1: cumulative sequence. BlobToolKit cumulative sequence plot. The grey line shows cumulative length for all scaffolds. Coloured lines show cumulative lengths of scaffolds assigned to each phylum using the buscogenes taxrule. An interactive version of this figure is available at https://blobtoolkit.genomehubs.org/view/ilAbrTrip1.1/dataset/CAJPHX01/cumulative.

**Figure 5.**
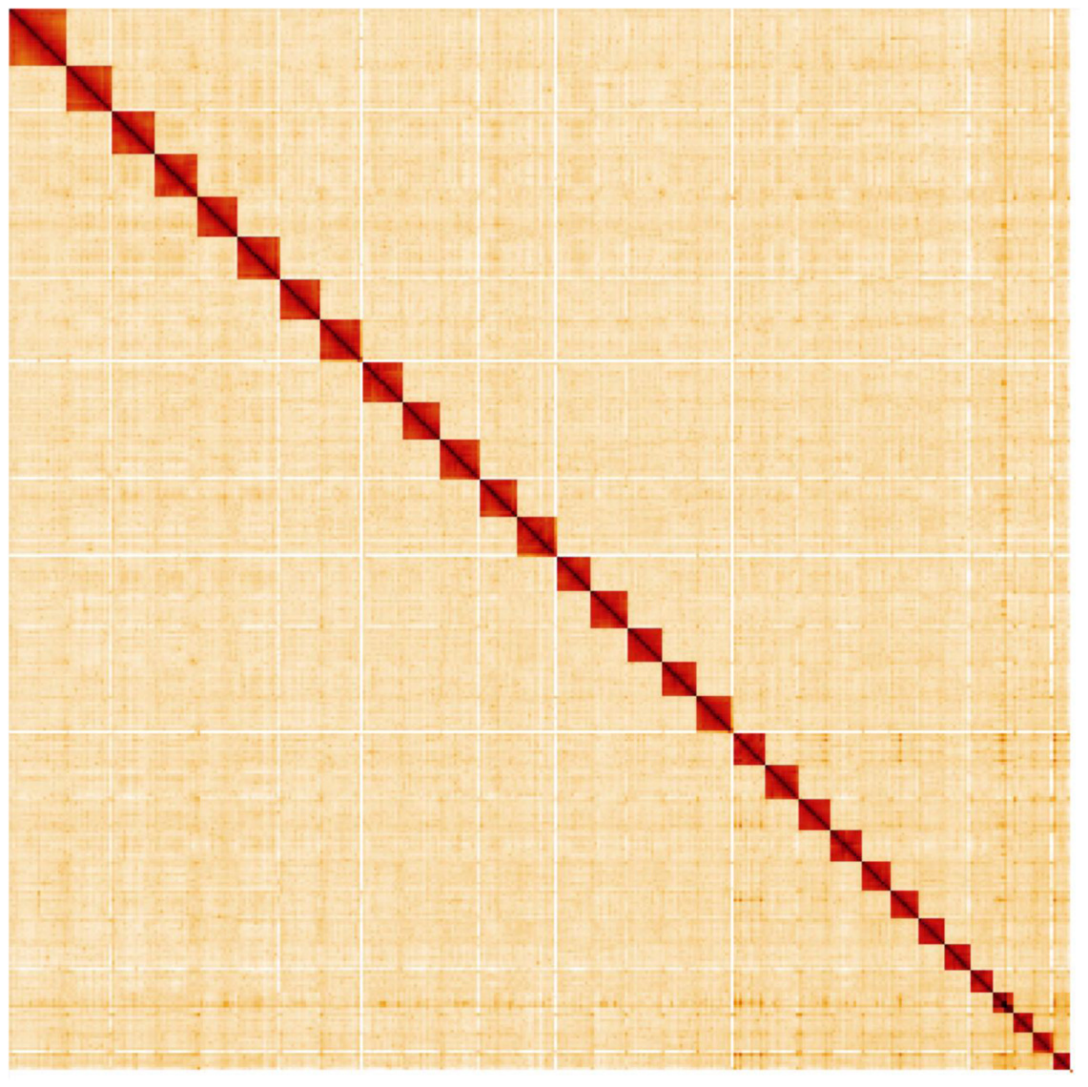
Genome assembly of *Abrostola tripartita*, ilAbrTrip1.1: Hi-C contact map. Hi-C contact map of the ilAbrTrip1.1 assembly, visualised in HiGlass.

**Table 2.** Chromosomal pseudomolecules in the genome assembly of *Abrostola tripartita*, ilAbrTrip1.1.

INSDC accession	Chromosome	Size (Mb)	GC%
HG996487.1	1	15.98	36.7
HG996488.1	2	15.59	36.8
HG996489.1	3	15.22	37
HG996490.1	4	15.13	36.7
HG996491.1	5	14.91	36.8
HG996492.1	6	14.74	36.3
HG996493.1	7	14.47	36.5
HG996494.1	8	14.45	36.3
HG996495.1	9	14.04	36.6
HG996496.1	10	13.84	36.3
HG996497.1	11	13.71	36.7
HG996498.1	12	13.65	36.3
HG996499.1	13	13.10	36.7
HG996500.1	14	12.99	36.7
HG996501.1	15	12.52	36.5
HG996502.1	16	12.52	36.9
HG996503.1	17	12.43	37.2
HG996504.1	18	12.03	37.2
HG996505.1	19	11.91	37.5
HG996506.1	20	11.64	37.1
HG996507.1	21	11.29	37.3
HG996508.1	22	10.01	37.4
HG996509.1	23	9.94	37.8
HG996510.1	24	9.43	37.3
HG996511.1	25	8.92	37.2
HG996512.1	26	8.52	37.5
HG996513.1	27	7.37	40.5
HG996514.1	28	7.24	38.7
HG996515.1	29	6.58	39.5
HG996516.1	30	5.91	39.2
HG996486.1	Z	20.96	36.4
HG996517.1	MT	0.02	19.9
-	Unplaced	0.03	38.5

## Methods

A single male *A. tripartita* (ilAbrTrip1) was collected from Wytham Woods, Oxfordshire (biological vice-county: Berkshire), UK (latitude 51.769, longitude -1.339) by Douglas Boyes, UKCEH, using a light trap. The sample was identified by the same individual, and preserved on dry ice.

DNA was extracted at the Tree of Life laboratory, Wellcome Sanger Institute. The ilAbrTrip1 sample was weighed and dissected on dry ice with tissue set aside for Hi-C and RNA sequencing. Thorax tissue was cryogenically disrupted to a fine powder using a Covaris cryoPREP Automated Dry Pulveriser, receiving multiple impacts. Fragment size analysis of 0.01-0.5 ng of DNA was then performed using an Agilent FemtoPulse. High molecular weight (HMW) DNA was extracted using the Qiagen MagAttract HMW DNA extraction kit. Low molecular weight DNA was removed from a 200-ng aliquot of extracted DNA using 0.8X AMpure XP purification kit prior to 10X Chromium sequencing; a minimum of 50 ng DNA was submitted for 10X sequencing. HMW DNA was sheared into an average fragment size between 12–20 kb in a Megaruptor 3 system with speed setting 30. Sheared DNA was purified by solid-phase reversible immobilisation using AMPure PB beads with a 1.8X ratio of beads to sample to remove the shorter fragments and concentrate the DNA sample. The concentration of the sheared and purified DNA was assessed using a Nanodrop spectrophotometer and Qubit Fluorometer and Qubit dsDNA High Sensitivity Assay kit. Fragment size distribution was evaluated by running the sample on the FemtoPulse system.

RNA was extracted from abdomen tissue in the Tree of Life Laboratory at the WSI using TRIzol (Invitrogen), according to the manufacturer’s instructions. RNA was then eluted in 50 μl RNAse-free water and its concentration assessed using a Nanodrop spectrophotometer and Qubit Fluorometer using the Qubit RNA Broad-Range (BR) Assay kit. Analysis of the integrity of the RNA was done using Agilent RNA 6000 Pico Kit and Eukaryotic Total RNA assay.

### Sequencing

Pacific Biosciences HiFi circular consensus and 10X Genomics Chromium read cloud sequencing libraries were constructed according to the manufacturers’ instructions. Poly(A) RNA-Seq libraries were constructed using the NEB Ultra II RNA Library Prep kit. Sequencing was performed by the Scientific Operations core at the Wellcome Sanger Institute on Pacific Biosciences SEQUEL II (HiFi), Illumina HiSeq X (10X) and Illumina HiSeq 4000 (RNA-Seq) instruments. Hi-C data were generated from head tissue using the Arima Hi-C+ kit and sequenced on HiSeq X.

### Genome assembly

Assembly was carried out with Hifiasm ( Cheng *et al.*, 2021); haplotypic duplication was identified and removed with purge_dups ( Guan *et al.*, 2020). One round of polishing was performed by aligning 10X Genomics read data to the assembly with longranger align, calling variants with freebayes ( Garrison & Marth, 2012). The assembly was then scaffolded with Hi-C data ( Rao *et al.*, 2014) using SALSA2 ( Ghurye *et al.*, 2019). The assembly was checked for contamination and corrected using the gEVAL system ( Chow *et al.*, 2016) as described previously ( Howe *et al.*, 2021). Manual curation ( Howe *et al.*, 2021) was performed using gEVAL, HiGlass ( Kerpedjiev *et al.*, 2018) and Pretext. The mitochondrial genome was assembled using MitoHiFi ( Uliano-Silva *et al.*, 2021). The genome was analysed and BUSCO scores generated within the BlobToolKit environment ( Challis *et al.*, 2020). Table 3 contains a list of all software tool versions used, where appropriate.

**Table 3.** Software tools used.

Software tool	Version	Source
Hifiasm	0.142	Cheng *et al.*, 2021
purge_dups	1.2.3	Guan *et al.*, 2020
SALSA2	2.2	Ghurye *et al.*, 2019
longranger align	2.2.2	https://support.10xgenomics.com/genome-exome/software/pipelines/latest/advanced/other-pipelines
freebayes	1.3.1-17-gaa2ace8	Garrison & Marth, 2012
MitoHiFi	1.0	Uliano-Silva *et al.*, 2021
gEVAL	N/A	Chow *et al.*, 2016
HiGlass	1.11.6	Kerpedjiev *et al.*, 2018
PretextView	0.1.x	https://github.com/wtsi-hpag/PretextView
BlobToolKit	2.6.2	Challis *et al.*, 2020

## Ethics/compliance issues

The materials that have contributed to this genome note have been supplied by a Darwin Tree of Life Partner. The submission of materials by a Darwin Tree of Life Partner is subject to the Darwin Tree of Life Project Sampling Code of Practice. By agreeing with and signing up to the Sampling Code of Practice, the Darwin Tree of Life Partner agrees they will meet the legal and ethical requirements and standards set out within this document in respect of all samples acquired for, and supplied to, the Darwin Tree of Life Project. Each transfer of samples is further undertaken according to a Research Collaboration Agreement or Material Transfer Agreement entered into by the Darwin Tree of Life Partner, Genome Research Limited (operating as the Wellcome Sanger Institute), and in some circumstances other Darwin Tree of Life collaborators.

## Data availability

European Nucleotide Archive: Abrostola tripartita (the spectacle). Accession number PRJEB43740; https://identifiers.org/ena.embl/PRJEB43740.

The genome sequence is released openly for reuse. The *A. tripartita* genome sequencing initiative is part of the Darwin Tree of Life (DToL) project. All raw sequence data and the assembly have been deposited in INSDC databases. The genome will be annotated using the RNA-Seq data and presented through the Ensembl pipeline at the European Bioinformatics Institute. Raw data and assembly accession identifiers are reported in Table 1.

## Author information

Members of the University of Oxford and Wytham Woods Genome Acquisition Lab are listed here: https://doi.org/10.5281/zenodo.4789929.

Members of the Darwin Tree of Life Barcoding collective are listed here: https://doi.org/10.5281/zenodo.4893704.

Members of the Wellcome Sanger Institute Tree of Life programme collective are listed here: https://doi.org/10.5281/zenodo.5377053.

Members of Wellcome Sanger Institute Scientific Operations: DNA Pipelines collective are listed here: https://doi.org/10.5281/zenodo.4790456.

Members of the Tree of Life Core Informatics collective are listed here: https://doi.org/10.5281/zenodo.5013542.

Members of the Darwin Tree of Life Consortium are listed here: https://doi.org/10.5281/zenodo.4783559.
