# Supplementary material for: The genome sequence of the spectacle, Abrostola tripartita Hufnagel, 1766
Source: Wellcome Open Res. 2021 Dec 6;6:330. [Version 1] doi: 10.12688/wellcomeopenres.17355.1 (PMC8753578; doi:10.12688/wellcomeopenres.17355.1)
Supplement: Peer review history

# Peer review: Reviewer response for version 1

**DOI:** 10.21956/wellcomeopenres.19185.r47467

**Published:** 2022-01-25

**Authors:** Uma Rao

**Affiliations:** 1 Division of Nematology, ICAR- Indian Agricultural Research Institute, New Delhi, Delhi, India

The paper is an announcement and is written succinctly. All methods are adequate and results are sufficient to explain the findings. It is recommend for indexing with one minor edit:

 There is one mismatch between data in text and figure. Table 1 and text report the longest scaffold as 16.0 Mb, whereas figure 2 shows it as 21 Mb, which should be corrected.

Are sufficient details of methods and materials provided to allow replication by others?

Yes

Is the rationale for creating the dataset(s) clearly described?

Yes

Are the datasets clearly presented in a useable and accessible format?

Yes

Are the protocols appropriate and is the work technically sound?

Yes

Reviewer Expertise:

Plant nematode interactions, genomics, transgenics

I confirm that I have read this submission and believe that I have an appropriate level of expertise to confirm that it is of an acceptable scientific standard.

# Peer review: Reviewer response for version 1

**DOI:** 10.21956/wellcomeopenres.19185.r47462

**Published:** 2022-01-11

**Authors:** Xingtan Zhang

**Affiliations:** 1 State Key Laboratory of Ecological Pest Control for Fujian and Taiwan Crops, Institute of Applied Ecology, College of Plant Protection, Fujian Agriculture and Forestry University, Fuzhou, China

The authors provide a high-quality chromosome-scale genome assembly of *Abrostola tripartita*. I believe these resources will be useful for the community. I only have two comments on this manuscript:

1. The authors used Arima Hi-C+ kit to construct the Hi-C but provide limited methods. What is the difference between Arima Hi-C and other related Hi-C methods? Similarly, the SALSA scaffolder was used without details. More information should be added.
2. The quality of genome assembly was only accessed using BUSCO and heatmap. I recommend adding more analysis to valid the genome assembly. For instance, the authors could map sequencing reads to their assembly and check the distribution of coverage along each chromosome.

Are sufficient details of methods and materials provided to allow replication by others?

Partly

Is the rationale for creating the dataset(s) clearly described?

Yes

Are the datasets clearly presented in a useable and accessible format?

Yes

Are the protocols appropriate and is the work technically sound?

Yes

Reviewer Expertise:

Genomics

I confirm that I have read this submission and believe that I have an appropriate level of expertise to confirm that it is of an acceptable scientific standard.

# Peer review: Reviewer response for version 1

**DOI:** 10.21956/wellcomeopenres.19185.r47465

**Published:** 2022-01-05

**Authors:** Youngik Yang

**Affiliations:** 1 National Marine Biodiversity Institute of Korea, Janghang-eup, South Korea

The data note by Boyes *et al.* presented a high-quality genome assembly of *Abrostola tripartita*, a grey and white noctuid moth. They applied state-of-the-art sequencing technologies and bioinformatics methods. The resulting assembly contained 32 pseudo-chromosomes and BUSCO was 99%. I believe that this high-quality chromosome-level assembly will be a valuable resource for further studies of this species as well as comparative studies with other species.

 I have some recommendations for improving the article.

1. Specimen image would be better to be shown next to the ruler not the FluidX storage. 
2. Please, provide the exact number of chromosomes and references if available. 
3. MitoHiFi requires a close-related species for input. Please, provide what it was. 
4. Figure 2 and 3 are not very informative in my opinion. The information in the figures can be combined in table 2. 

Are sufficient details of methods and materials provided to allow replication by others?

Yes

Is the rationale for creating the dataset(s) clearly described?

Yes

Are the datasets clearly presented in a useable and accessible format?

Yes

Are the protocols appropriate and is the work technically sound?

Yes

Reviewer Expertise:

Bioinformatics, Genomics

I confirm that I have read this submission and believe that I have an appropriate level of expertise to confirm that it is of an acceptable scientific standard.
